# Functional MRI of murine olfactory bulbs at 15.2T reveals characteristic activation patterns when stimulated by different odors

**DOI:** 10.1038/s41598-023-39650-0

**Published:** 2023-08-16

**Authors:** Odélia Chitrit, Qingjia Bao, Aoling Cai, Silvia Gabriela Chuartzman, Noga Zilkha, Rafi Haddad, Tali Kimchi, Lucio Frydman

**Affiliations:** 1 Department of Chemical and Biological Physics, Weizmann Institute of Science, Rehovot, Israel; 2 Department of Brain Sciences, Weizmann Institute of Science, Rehovot, Israel; 3 Innovation Academy for Precision Measurement Science and Technology, Chinese Academy of Sciences, Wuhan, China; 4 The Gonda Multidisciplinary Brain Research Center, Bar-Ilan University, Ramat-Gan, Israel

**Keywords:** Olfactory system, Functional magnetic resonance imaging, Magnetic resonance imaging

## Abstract

Thanks to its increased sensitivity, single-shot ultrahigh field functional MRI (UHF fMRI) could lead to valuable insight about subtle brain functions such as olfaction. However, UHF fMRI experiments targeting small organs next to air voids, such as the olfactory bulb, are severely affected by field inhomogeneity problems. Spatiotemporal Encoding (SPEN) is an emerging single-shot MRI technique that could provide a route for bypassing these complications. This is here explored with single-shot fMRI studies on the olfactory bulbs of male and female mice performed at 15.2T. SPEN images collected on these organs at a 108 µm in-plane resolution yielded remarkably large and well-defined responses to olfactory cues. Under suitable T2* weightings these activation-driven changes exceeded 5% of the overall signal intensity, becoming clearly visible in the images without statistical treatment. The nature of the SPEN signal intensity changes in such experiments was unambiguously linked to olfaction, via single-nostril experiments. These experiments highlighted specific activation regions in the external plexiform region and in glomeruli in the lateral part of the bulb, when stimulated by aversive or appetitive odors, respectively. These strong signal activations were non-linear with concentration, and shed light on how chemosensory signals reaching the olfactory epithelium react in response to different cues. Second-level analyses highlighted clear differences among the appetitive, aversive and neutral odor maps; no such differences were evident upon comparing male against female olfactory activation regions.

## Introduction

Social behavior and communication among mammals rely heavily on olfactory cues. In humans olfactory functions play a vital role in daily life, shaping emotions, memories and actions; odor identification and deficits are also early markers in neuropsychiatric and neurodegenerative diseases^1,2^. In rodents, and more specifically in mice, this sense is of paramount importance. It assists in mating choices, location of desirable items, distinction between individuals, territorial marking, predator and danger avoidance, and the determination of health status^3^. There are striking similarities between species in the organization of the olfactory pathway in all these processes^4^. Therefore, preclinical studies are of key importance for advancing an understanding of the olfactory processes, and of its many behavioral consequences. Studying the neural activation of olfaction in living brains with minimal invasiveness is enabled by functional techniques, foremost among them by in vivo functional Magnetic Resonance Imaging (fMRI)^5–7^. fMRI’s potential, however, is hard to fully realize in functional olfactory studies on mice. Indeed, the detection of the Blood Oxygenation Level Dependent (BOLD) contrast underlying fMRI^8^, requires single-shot imaging techniques^9–11^ as well as high sensitivity—particularly when targeting small organs like the olfactory bulb (OB). This organ, mediating the transmission of olfactory information between the exterior and the brain, lies close to the nose and eyes; organs proximate to air interfaces, which subject nearby tissues to field inhomogeneities. The distortions and susceptibility artifacts associated to these inhomogeneities increase when using the ultrahigh field (UHF) methods that can boost a much-needed sensitivity—and particularly single-shot methods like echo planar imaging (EPI), underlying most of fMRI^12–14^. Functional studies have thus been demonstrated on large rodents like rats subjected to odor stimulation^15–19^, but accurate activation maps on mice—with their treasure trove of physiological function and behavioral responses associated to genetically engineered models—remain elusive^20,21^.

This study explores the potential of UHF fMRI based on SPatiotemporally ENcoding (SPEN) acquisition sequences^22–24^, customized for studying neural activations of OB in mice. SPEN is a single-shot 2D MRI technique that can significantly reduce the distortions affecting EPI, particularly if executed in a so-called fully refocused mode canceling the inhomogeneous (T2*) broadenings affecting the signal^25–27^. In this study, male and female mice were exposed to neutral, appetitive or aversive odors at different concentrations under suitable anesthetic protocols, and their OBs were subject to SPEN-based fMRI analyses at 15.2 T using cryogenically cooled receiving coils for further sensitivity. Intensity and spatial analyses of the neural responses were obtained for different odors, enabling comparisons between sexes, stimuli, concentrations, as well as dual- vs. single-nostril control experiments. Experiments were also compared to more traditional echo-planar-imaging (EPI) fMRI OB counterparts, whose performance at these UHF was found lacking. Second-level analyses of the SPEN data revealed clear differences among the spatial regions activated by the different odors, yet no distinctions between the OB regions activated in male and female animals were observed. The latter, however, evidenced statistically significant lower values.

## Methods

### Animal preparation and anesthesia protocols

**Figure 1:**
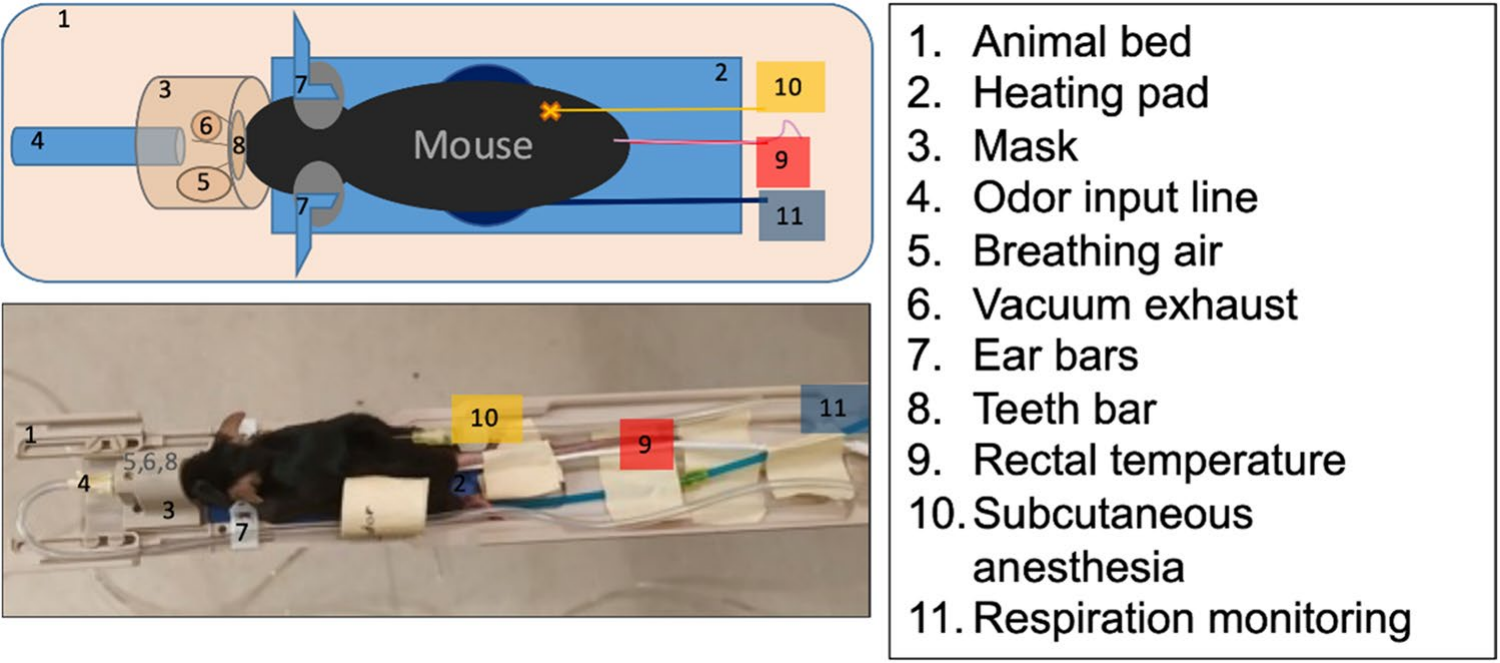
Scheme and photograph of the animal set up used in the present study.

All animal procedures were approved by the Institutional Animal Care and Use Committee of the Weizmann Institute of Science, which is fully accredited by the AAALAC, the US NIH Office of Laboratory Animal Welfare, and the Israel Ministry of Health. All methods and procedures were performed in accordance to relevant guidelines and regulations. This study is reported in accordance with ARRIVE (Animal Research: Reporting of In Vivo Experiments) guidelines. Animals were housed in cages in a 12 h daylight/12 h night cycle, with water and food available ad libitum. In total, 38 adult C57BL/6 mice (Envigo, Jerusalem) with a minimum age of 13 weeks, were studied. These included 30 males and 8 females, split as follows: n = 10 males for neutral odor experiments, n = 5 males for varying concentration experiments, n = 3 males for single nostril experiments, n = 7 males for aversive stimulation experiments, n = 6 males for appetitive stimulation experiments, and n = 8 females for sex comparison with neutral stimulation experiments. A number of protocols employing different anesthetics to keep the mice stable, with suitable respiration rate but also meaningful functional response, were tested; in the end, the protocol of Zhao et al.^28^ was adopted. Here animals are initially anesthetized with isoflurane during 20 min (3% isofluorane for induction and 2% during set-up), followed by a first subcutaneous bolus injection of 0.2 mg/kg medetomidine. Animals are then secured in a prone position in a cradle, with their head restrained with tooth and ear bars to reduce motion (see Fig. 1). Medetomidine is thereafter infused continuously at a rate of 0.6 mg/kg/h via an IV catheter, and medical air (a mixture of 80% N_2_ and 20% O_2_) delivered to the animal via a nose-cone throughout the experiment. Isoflurane is progressively decreased after starting the medetomidine infusion so as to keep the respiration rate constant, and eventually stopped. At the end of each study, the mouse recovers by a subcutaneous injection of medetomine’s antisedative (atipamezole hydrochloride ~ 1.0 mg/kg). Temporary nare occlusion experiments were performed by plugging with medical cotton, paraffin and glue, blocking one of the animal’s nasal cavities.

### Olfactory and fMRI procedures

fMRI experiments were performed using a custom-made olfactometer constructed for an odor stimulation of the animals. This device is equipped with 8 valves whose opening/closing is controlled by a data acquisition card, programable via a customized GUI written in Matlab (The Mathworks Inc.) which can be synchronized with the MRI acquisition. The olfactory stimulants included isoamyl acetate (banana smell) as a neutral odor, 2,3,5-trimethyl-3-thiazoline (TMT—‘fox’ smell) as an aversive odor, and 2-phenylethanol (PEA—floral smell) as appetitive odor^29^. Isoamyl acetate was adopted as neutral odor based on previous rat-oriented fMRI studies^30,31^, even if it has been recently suggested that amyl acetate—a related chemical—could induce stress or analgesia in male rodents^32^. A number of different odor delivery protocols including different mouse masks, tubings, vacuum/pressures, etc., were tested, in the search of an optimal compromise among parameters that would lead to a reliable fMRI protocol. All these tests were done under neutral odor stimulation. In the end, the adopted setup included a joint supply of air and odor mixture, controlled and delivered through separate channels to a nose-cone that included a third, return line. The return line was connected to the mask and a weak vacuum was continuously applied on it, in order to swiftly evacuating the odors. To deliver the odors air was passed through Telflon^®^ bottles equipped with disposable sponges, in which the odorants were adsorbed with the aid of mineral oil. The odor’s concentration was measured at the outlet of the evacuating line with a photoionization detector (ppbRAE 3000 system, Honeywell) during the course of each fMRI acquisition; odors in this detector achieved steady states within ca. 10 s following the opening of the valves. All channels/tubes and animal masks were cleaned with alcohol before each experiment in order to avoid contaminations. Between different odor tests, all the tubes and connectors were replaced. The final paradigm chosen for this study involved the repetition of 10 events involving 120 s of air flow, followed by 26 s of odor flow. These times were chosen to ensure the full cleaning of the lines between the odor stimulations, and the achievement of a robust functional response as visible by simple inspection of the signal intensities in the processed images. Shorter odor stimulation times failed to provide consistent systematic responses (consider that the first 10 s include the system’s ramp up time); odor stimulation times ≥ 40 s evidenced signal decrease due to adaptation/saturation. Each full fMRI examination was therefore ≈ 23 min long, and involved 700 scan repetitions. At least two such runs were performed for the animals whose data is here considered; up to four runs were performed when animal stability permitted it. Multiple comparison corrections were performed in the statistical analyses. Control experiments were also run in each examination using the same set up and protocol, but flowing air through both the breathing and odor channels feeding into the mask.

An SA-II small animal system was used to monitor and control the animal physiology during the preliminary setting up stage as well as throughout the fMRI data acquisitions, which started 20 min after inserting the animals in the magnet to allow for good respiration and temperature stabilization (140 ± 40 breaths per minute; 36 ± 0.5 °C rectal temperature). Fluctuations in the females respiration rates were more marked than on the males: ca. ± 50 breaths/min vs. ± 30, respectively; this could account for the different motional artifacts of the two cohorts, leading to a need to discard a larger proportion of the female fMRI data (vide infra). During these pre-fMRI periods all MRI calibrations were performed, including the acquisition of high resolution anatomical images with Rapid-Acquisition-with-Relaxation-Enhancement (RARE) sequence for anatomical purposes, as well as shimming procedures.

MRI experiments were performed on a horizontal Biospec® 15.2 Tesla Ultrashielded™ preclinical scanner equipped with an Avance IIIHD console, a B-GA 6S-100 3-axis gradient system with a 60 mm inner diameter capable of delivering a gradient strength of 1000 mT/m, and integrated 2nd and 3rd order shim sets. A number of surface and volume coils were assayed on this system; in the end, fMRI acquisitions were performed using a surface ^1^H quadrature transmit/receive CryoProbe® surface coil with an inner diameter of 20 mm (tests were also carried out using non-cryogenic surface and volume coils, but their results are not presented here as the data Signal Noise Ratio (SNR) was substantially lower than with the cryocoil).

**Figure 2:**
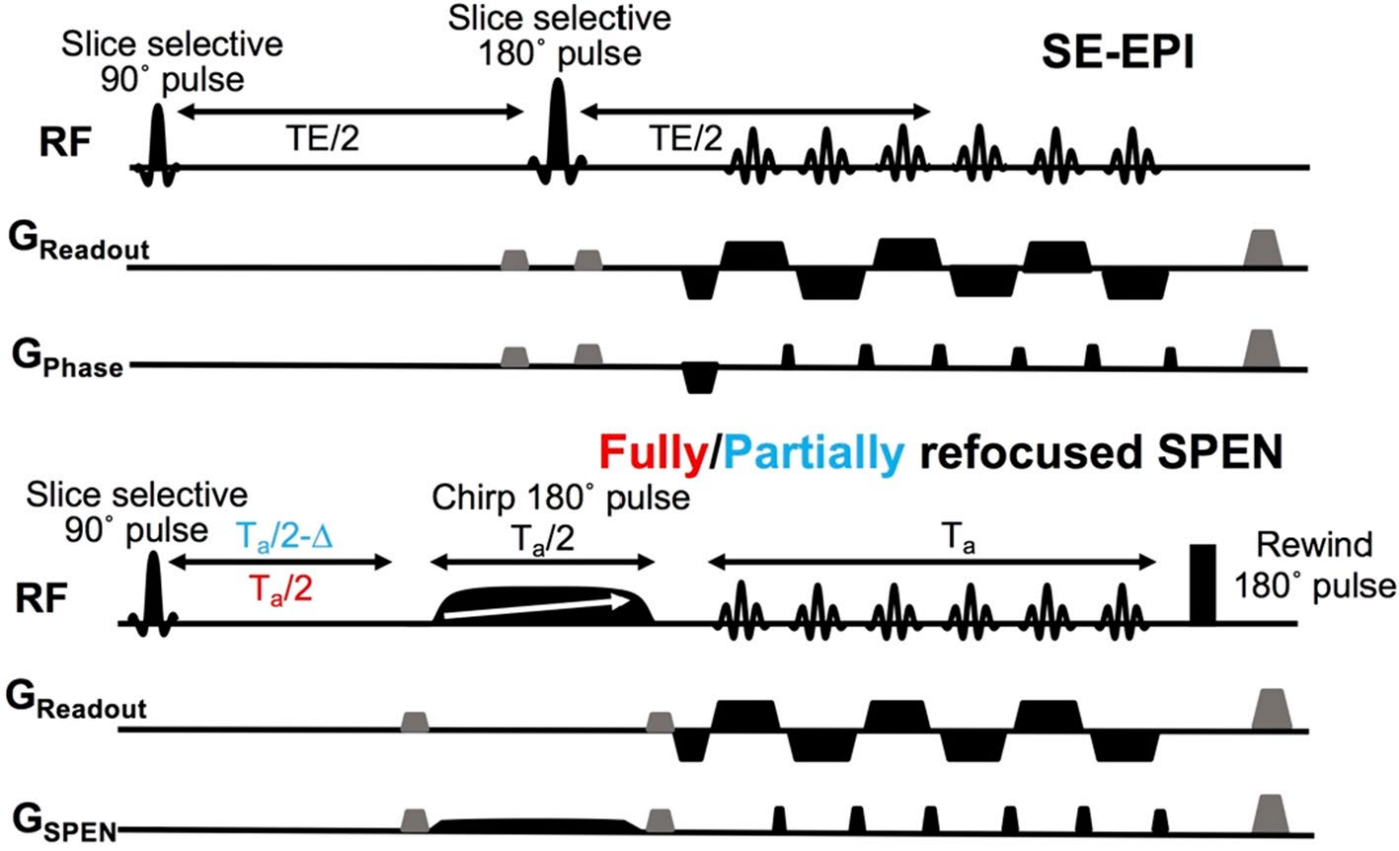
Schemes of single-shot 2D SE EPI and SPEN sequences used to scan OBs in mice. SPEN includes a 180° chirp pulse acting in the presence of a gradient that encodes the more artifact-prone, low bandwidth dimension with a quadratic phase, a pre-encoding delay whose duration can achieve full/partial refocusing of T2* effects—including BOLD effects from macroscopic vessels, and a final broadband “rewind” 180° pulse bringing back to equilibrium all non-excited slices. Timings illustrate SPEN experiments collected under full-refocusing (red) and partial-refocusing (cyan) conditions. Gradients shaded in black are performing an encoding; those in gray are purging and crushing gradients. For simplicity the figure obviates the slice-selective gradient.

Four kind of single-shot fMRI experiments were assayed. These included gradient-echo and spin-echo (GE and SE) EPI single-shot sequences^10,11^ supplied by Bruker (Fig. 2, top); and two 2D versions of SPEN MRI incorporating fully-refocused and partially-refocused (FR and PR) timings in their sequences (Fig. 2, bottom)^24,25^. Figure 2 illustrates the main differences between the SPEN sequences and SE-EPI; further parameters about these experiments are provided in Supplementary Information Table S1. Notice that the SPEN experiment incorporates a frequency-swept (chirped) pulse that, applied in unison with a gradient, imparts a quadratic phase on the spins along their encoding direction. This leads to a subsequent imaging of the targeted region along this axis directly in the spatial domain as read out by a blipped gradient over the course of the acquisition time T—without the need for a Fourier transform. SPEN’s avoidance of a Fourier analysis allows one to tailor the bandwidth of its spatial dimension to the field inhomogeneities are active, thereby overcoming the latter’s effects and improving image robustness. While this is done at the expense of sensitivity, the latter can be regained by using super-resolution algorithms^33–35^, that compensate in part for the absence of Fourier’s multiplexing advantages. Even further robustness can be achieved by timing SPEN’s delays, pulse lengths and acquisition times to so-called full-refocusing conditions (Fig. 2, top)^25,27,36^, under which each voxel will emit its signals with minimal inhomogeneous broadening effects throughout the course of the acquisition. While this attenuates the BOLD effect from large capillaries, it still leaves the effects of small microscopic vessels leading to T2-like dephasing effects. A final advantage derived from the non-Fourier nature of these sequences is an ability to zoom into the region of interest without folding—a feature that was here exploited to focus on the relatively small murine OBs, thus gaining in spatial resolution without suffering from foldover artifacts. A limitation of these SPEN experiments is their relatively high power deposition, which circumscribed the experiments to the acquisition of few (2 or 3) slices; given the small dimensions of the OBs, however, this sufficed for a full coverage. No significant differences were observed upon processing these different slices, and hence only one slice is generally reported. High power deposition together with sensitivity considerations also constrained the minimum repetition time TR that could be used in such experiments, which was set to 1 s. Another constraint arose from SPEN’s full refocusing condition, which sets a relatively long TE that needs to match the acquisition time (Fig. 2). All SPEN sequences employed in this study together with their reconstruction pipelines are available at: https://www.weizmann.ac.il/chemphys/Frydman_group/software.

### Anatomical determinations and fMRI signal analyses

**Figure 3:**
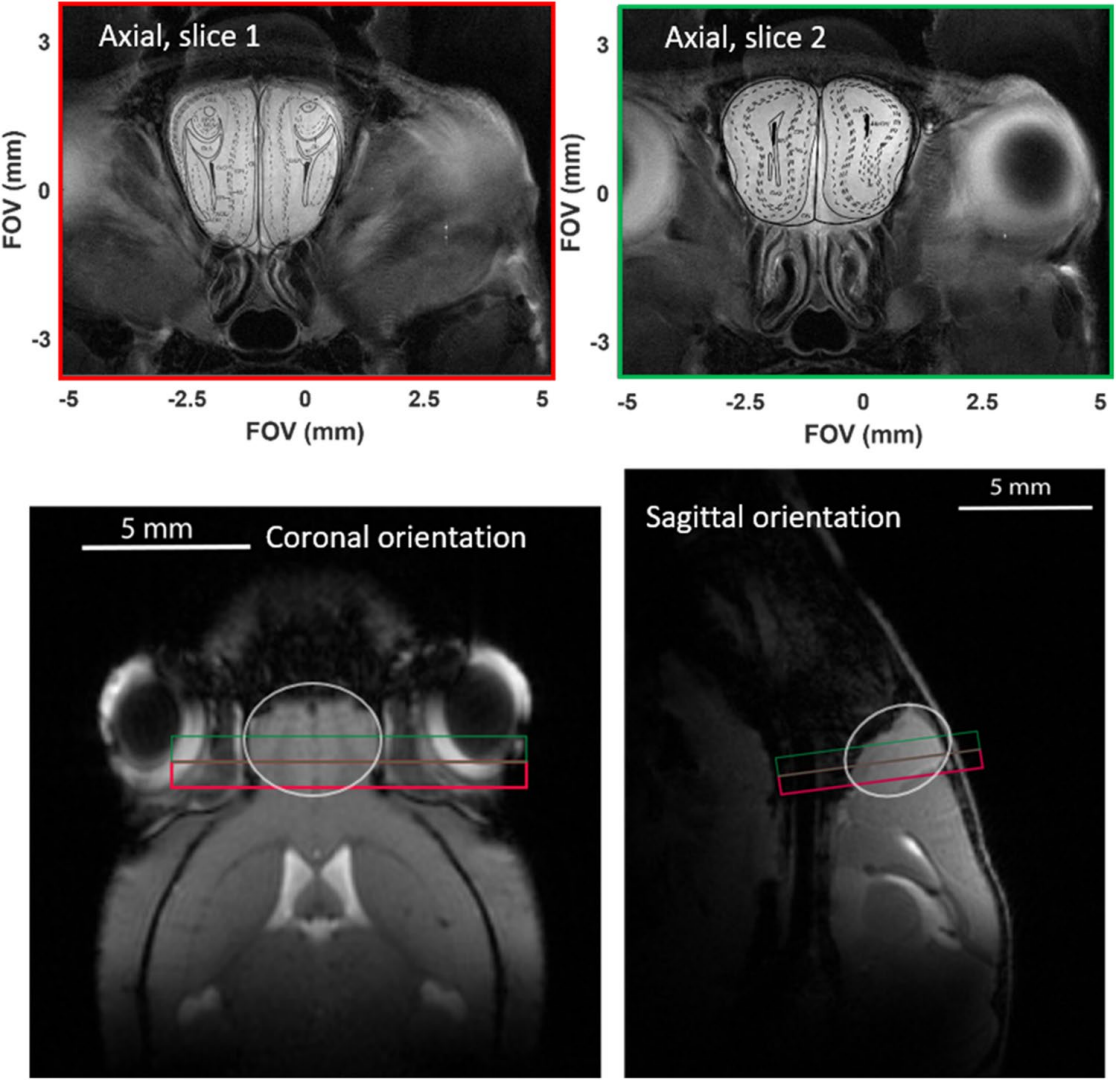
High resolution RARE images used to select the targeted OB slices: Axial slices with Paxinos—Franklin Atlas data superimposed on top; coronal and sagittal slices (with OB encircled in white) on the bottom. Images were collected at 15.2T with the following parameters: resolution = 30 × 30 µm^2^; echo spacing = 7.33 ms; RARE factor = 8; slice thickness = 0.8 mm; TR = 2.8 s; 4 averages; acquisition time = 6 m 54 s.

fMRI analyses relied on selecting the OB region of interest (ROI) or a part of the OB in specific cases, as guided by the Paxinos and Franklin brain Atlas^37^; Fig. 3 illustrates representative RARE MRI data used to define the targeted regions.

**Figure 4:**
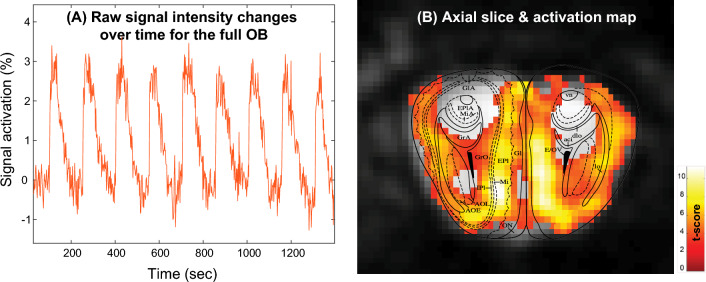
(**A**) Raw activation-derived MRI signal changes of an entire OB slice acquired with partially refocused SPEN and the stimulation protocol described in the text. (**B**) SPM fMRI map derived from (**A**) with a threshold value at *p* = 0.001, superimposed on the single-shot SPEN MR image and on an Atlas cut of the OB for this specific slice.

Initial assessments on the quality of the fMRI activation data were done by simply integrating the signal intensities of full OB images acquired every 2 s while under the stimulation paradigm, without any statistical treatment. Plotting these mean OB MR signal intensities throughout the course of the acquisition, enabled an assessment on the correctness of the protocol and its overall robustness with minimal statistical biases (Fig. 4A). Baseline drifts and events such as motions could also be assessed from this raw image processing; if/when the latter were too severe as assessed by these plain examination, experiments were discarded. This led to a relatively large number (≈ 50%) of the female experiments being discarded, either due to evident motions or due to uncertain activations in the raw images. By contrast, only ca. 15% of the male experiments (≈ 20% in the variable concentration and ≈ 12% in the variable odor tests) were discarded by these criteria.

Images showing this kind of clear intensity differences upon cycling between air flow and odor stimulation in the OB, were processed by the SPM software^38^ (Statistical Parametric Mapping, v. 12, Wellcome Trust Centre for Neuroimaging, London, UK). Retrieval of these activation maps included a Gaussian smoothing with a 2.5 mm kernel, slice realignment, and plotting at different threshold *p*-values and different t-test confidence levels (as indicated in the respective figures; e.g., Fig. 4B). Canonical Hemodynamic Response Functions (HRF) without derivatives with no Finite Impulse Response were used in the processing; a second order time-modulation of the HRF was assumed for accounting for non-stationary behaviors. Second-level analyses were also performed to evaluate differences among the stimulation maps elicited by the appetitive, aversive and neutral odor maps on males, as well as between the male and female maps arising for neutral odor activation. For each of these examined groups, three maps were first obtained from first-level one-way ANOVA analyses. For the between-group analyses, the contrasts were obtained as *t*-maps of differences between the average activation of each stimuli; these were calculated after each the images of each data set had been re-oriented (if needed), co-registered, and normalized to one another.

Second-level analyses were also performed to evaluate differences among the stimulation maps elicited by the appetitive, aversive and neutral odor maps on males (one-way ANOVA), as well as between the male and female maps arising for neutral odor activation (two-sample T-test). For the between-group analyses, the contrasts were obtained as *t*-maps of differences between the average activation of each stimuli; these were calculated after each the images of each data set had been re-oriented (if needed), co-registered, and normalized to one another. Given the relatively small number of samples included in these analyses (*n* = 3), these results were also evaluated using SnPM—a non-parametric statistical framework.

## Results

### Comparing acquisition methods

**Figure 5:**
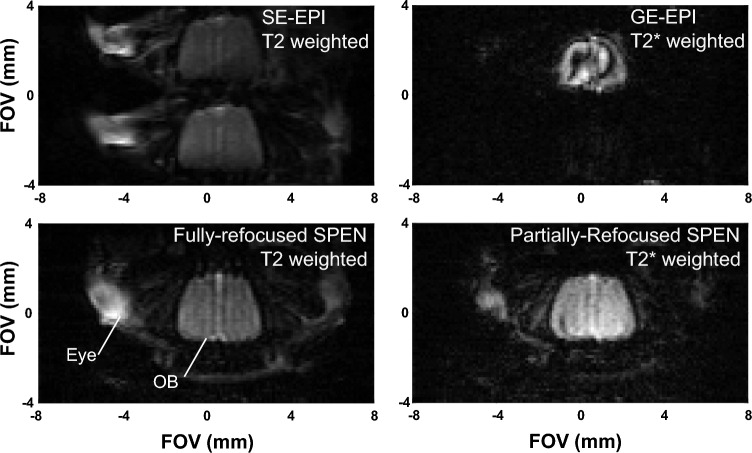
15.2T T2 and T2* weighted images of the OB collected with different EPI and SPEN variants. Notice the artifacts and image distortions arising from the SE EPI and GE EPI sequences respectively, when targeting at high fields this organ close to the eyes and to air interfaces. Although the best quality images arise from the FR SPEN experiments, full refocusing conditions were broken by introducing a delay Δ (cf. Figure 2) for the sake of enhancing the T2* weighting. For all images TR = 1 s; TE ≈ 26 ms; 1 segment; 2 averages; FOV = 10.4 × 7.5 × 0.8 mm^3^; nominal resolution = 108 × 107 × 800 μm^3^. Average SNR for the OB region in the SPEN images was 30 ± 10.

Figure 5 compares single-shot data collected using EPI- and SPEN-based acquisition methods, when focusing on a mouse OB region at 15.2T. The improvement in image quality as one progresses from GE to SE-EPI and onwards to partially- and fully-refocused SPEN, are evident. Thus, although in some cases both GE and SE EPI showed activation in the OB region (Supplementary Information, Fig. S1), difficulties in retrieving artifact-free, well-defined features for the OB from EPI experiments, led us to adopt the SPEN counterparts for further explorations. Furthermore, EPI experiments were more often affected by false activation problems, particularly when the olfactometer switched between the air and odor channels. This led to functional-like activation for regions surrounding the OB in paradigms that involved no odor, just a change of air from one pipe line to another (Supplementary Information, Fig. S2). The best image quality was usually provided by the fully-refocused SPEN acquisitions; notice that while avoiding spatial distortions, no sensitivity penalties are paid by these images (Supplementary Information, Table S1). Still, the full T2* refocusing involved in such experiments attenuated some of their potential to detect fMRI’s BOLD effects. Partially refocused SPEN including a net T2* weighting time Δ = 4 ms, were thus also used in a majority of the experiments.

### Single-nostril experiments

**Figure 6:**
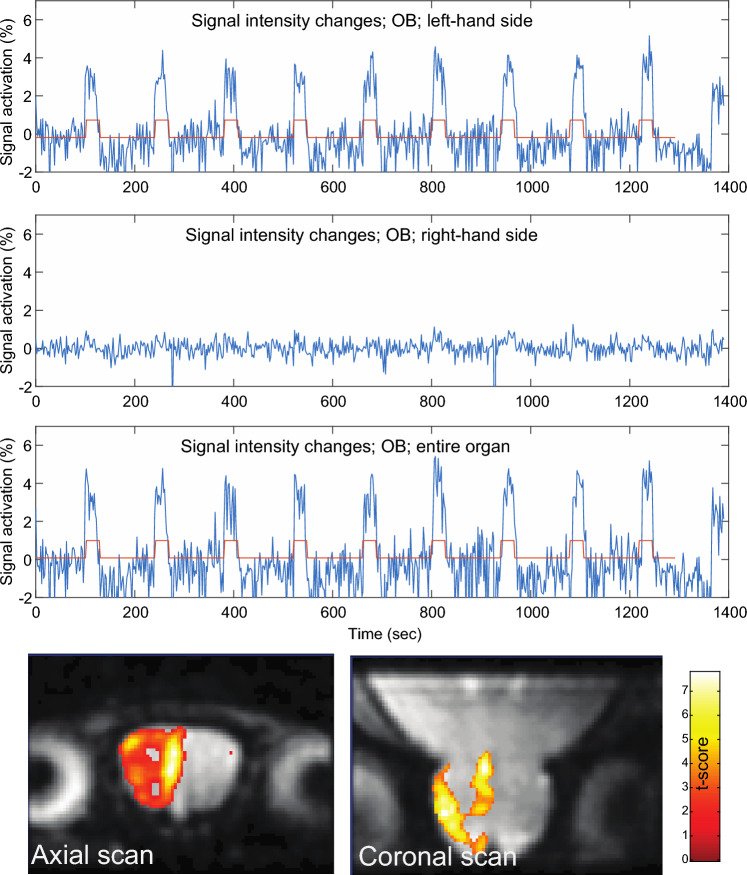
Raw fMRI signals (in blue) of the left, the right and of both OB hemispheres acquired upon sealing the left nostril of a stimulated mouse. Shown in red is the timing of the olfactory paradigm. Images were collected with a fully refocused SPEN sequence, using a neutral (isoamyl acetate) odor stimulation paradigm at 50 ppm concentration. Shown on the bottom are SPM fMRI maps for different runs on the same animal, plotted with a threshold value *p* = 0.001.

The signal changes shown in Fig. 4 clearly correlate with the odor paradigm. Still, a supplementary test control was carried out by relying on mice’s stereo-olfaction, whereby each hemisphere in the OB communicates with only one of the animal’s nostrils. A series of odor stimulation experiments were thus run after reversibly plugging up left and right nostrils of different mice. The impact of occluding nares in such fashion were clearly reflected on the activation signals and maps of the OB, with the clogging of the left-hand nostril leading to activation on the right-hand hemisphere of the OB, and vice versa (Fig. 6).

### Neutral odor activation at different concentrations

**Figure 7:**
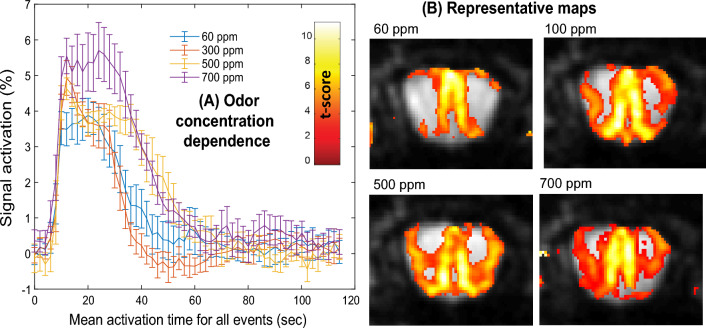
(**A**) Mean of the OB fMRI activations observed for n = 3 males, exposed to different concentrations (60, 300, 500 and 700 ppm) of a neutral odor. The odor stimulation (isoamyl acetate) lasted for 26 s; events were repeated every 120 s. (**B**) SPM maps deriving from different concentrations (*p* < 0.001 threshold) for a representative animal.

With the protocol’s reliability verified, the fMRI effects of different odor concentrations on the stimulation observed in the OB, was evaluated. As illustrated in Fig. 7 for a number of male animals, it appears that stronger effects in terms of spatial extent, intensity and duration of the OB activation, are observed with increasing odor concentrations. Still, the fMRI activation extent is markedly non-linear, and even small concentrations provide unambiguous activations extending throughout certain regions of the OB. It’s important to highlight that in general, physiological odors will not exceed 100 ppm^39^; these results suggest that a saturation of the olfactory response will occur past this threshold.

### OB activation and animal sex

**Figure 8:**
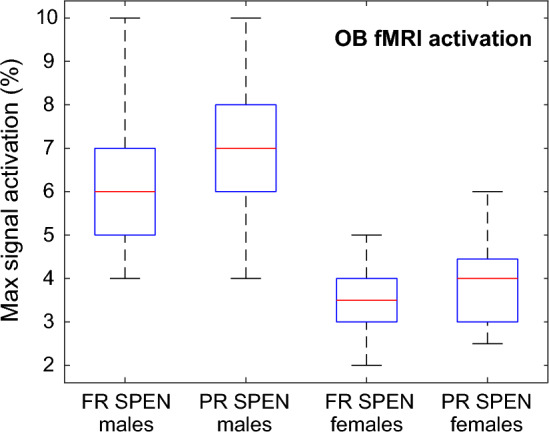
Box-whiskers plots describing the maximal signal activation observed for murine OB’s examined by fully- and partially-refocused SPEN sequences. Data for n = 10 males and n = 7 females are summarized, in all cases subject to an isoamyl acetate (neutral odor) stimulation at 450 ppm.

Figure 8 summarizes signal activation results obtained for male and female animals, subject to a protocol with neutral odor stimulation at a 550 ppm concentration. It follows from these data that although in both instances activation was strong, females present lower activation than males. Experimentally, it was also observed that female mice were sometimes less stable than males, in the sense of presenting more motion and less respiration rate stability during the examinations than male counterparts (see Experimental). As the same isofluorane setup protocol was used for male and female animals, and since females tend to be smaller than males, this may have had an impact on the stability of the fMRI experiments. Further research is in progress to clarify if lowering the isofluorane induction can also help increase the female signal activation amplitudes. Notice as well that, in general, the overall signal activation evidenced by the partially refocused sequence was slightly higher than in full refocused SPEN counterparts.

**Figure 9:**
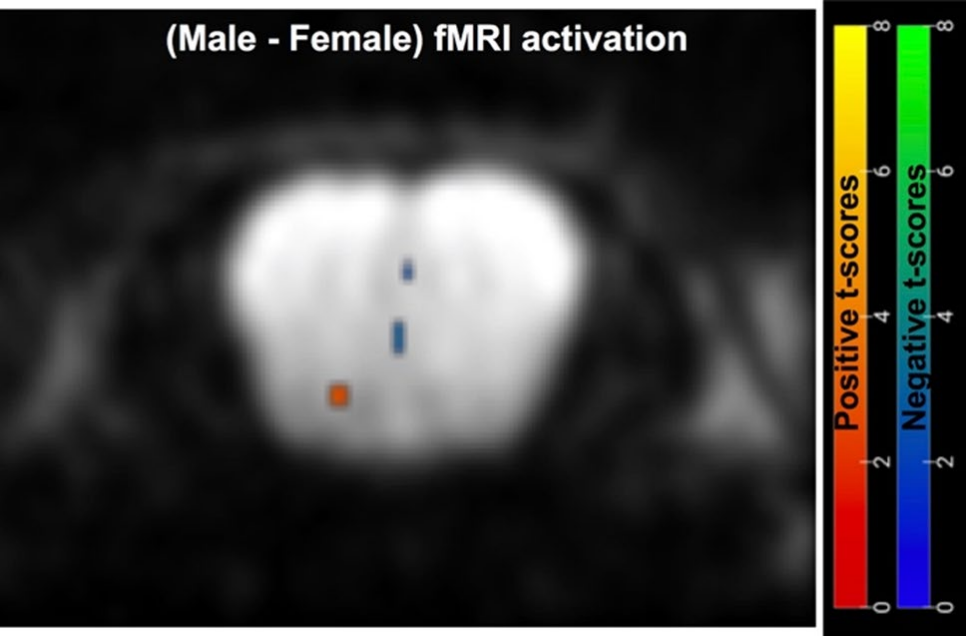
Second-level (SPM) difference map (*p* < 0.05) arising upon subtracting the average activations elicited by male and female cohorts (n = 3 animals each) scanned by PR SPEN fMRI, subjected to a 450 ppm isoamyl acetate (neutral) odor stimulation.

Despite the differences in activation intensities shown in Fig. 8, no significant differences in the OB regions activated in male and female mice could be detected. This is further evidenced by the SPM-derived two-sample t-test shown in Fig. 9, showing the difference between *n* = 3 animals scanned for each sex for equal isoamyl acetate stimulations at 550 ppm. Upon averaging these multiple scans for each sex, co-registering them and subtracting their normalized outcomes, no significant activation is visible.

### Changing the odor stimulation

**Figure 10:**
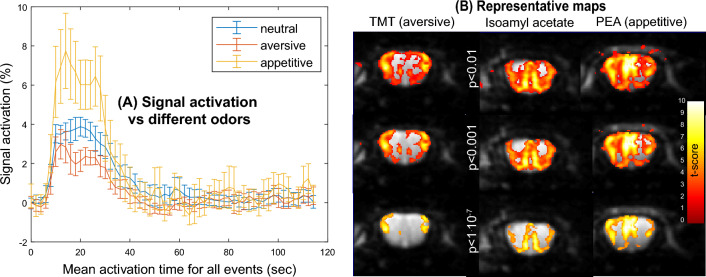
(**A**) Mean of the activation displayed by aversive (TMT), appetitive (PEA), and neutral (isoamyl acetate) odors, as arising from n = 3 animals/stimulations probed by PR SPEN fMRI. (**B**) SPM maps arising from the different odors for differing p-level thresholds, showing the average over the n = 3 animals for each odor.

**Figure 11:**
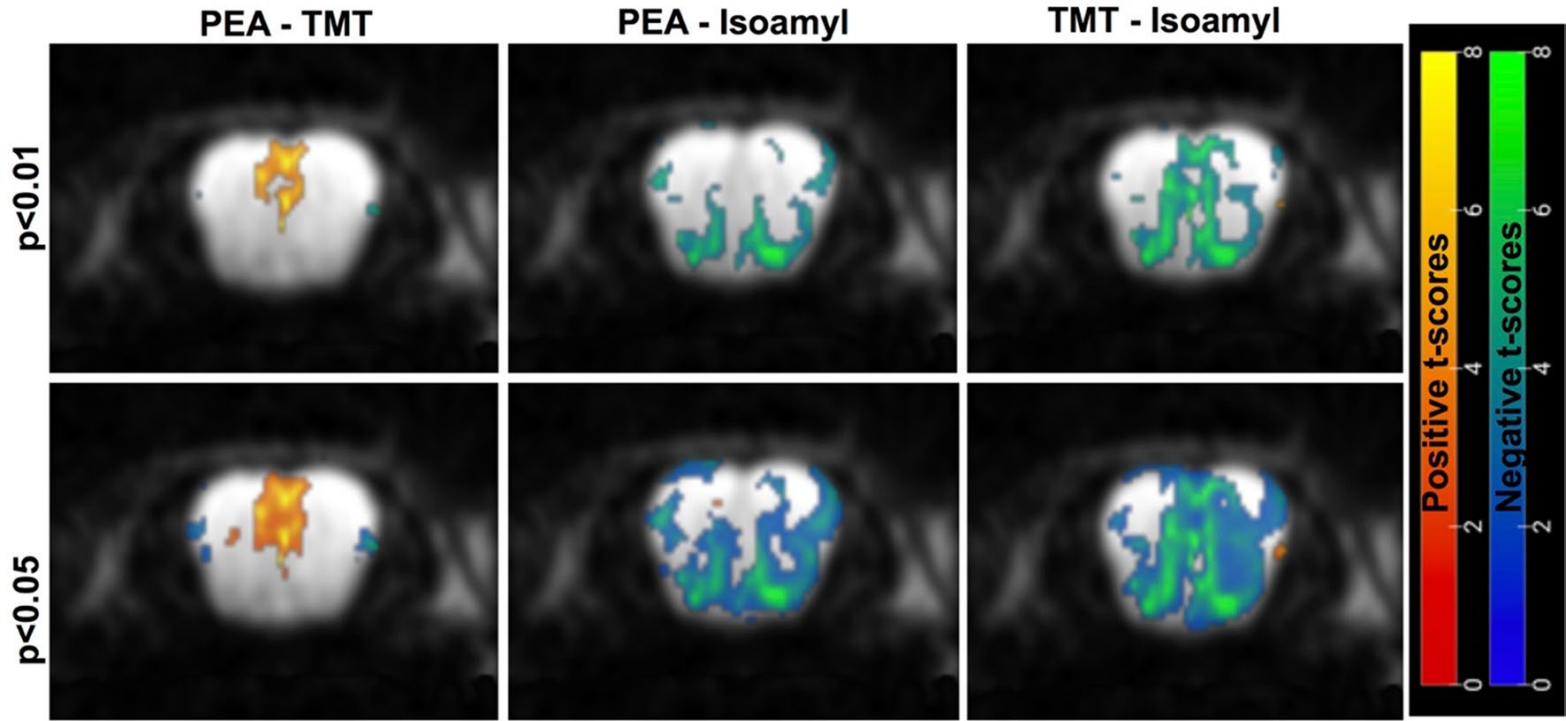
Second-level (SPM) analyses of the difference maps arising upon subtracting the averaged maps elicited by TMT (aversive), PEA (appetitive), and isoamyl acetate (neutral) odors; each of these as probed from n = 3 animals scanned by PR SPEN fMRI upon subjecting them to 60 ppm stimulations. These subtracted maps are shown for two p-level thresholds, and reveal both positive and negative t-scores reflecting stronger/weaker activation in these areas.

Figure 10 compares the mean signal activation and activation maps obtained when male mice were stimulated by different odors, applied using the same timing paradigm and at the same (60 ppm) concentration. The signal activation presents the highest amplitudes for appetitive and neutral odors. In fact, unstable motion and respiration rates were noticed during the aversive (TMT, mimicking a fox-like) odor stimulation: an average drop of ca. 20 breaths/min was noticed upon subjecting the anesthetized animals to aversive odors. Still, even in such instances, the signal and image qualities were good and allowed us to extract statistical (SPM) maps. Even at small *p-*value thresholds, these experiments highlight specific activation in the external plexiform region and in glomeruli in the lateral part of the bulb, when stimulated by aversive or appetitive odors, respectively. Neutral odor activations seemed to be sums of the regions activated by the appetitive and aversive stimulations. To further assess this hypothesis, second-level analyses of the partially refocused SPEN data were carried out for the average maps arising from the TMT, isoamyl acetate and PET odor stimulations. This assessment relied on *n* = 3 animal scans for each odor type; data from these multiple scans were re-oriented and co-registered as needed, and normalized among the different odors prior to subtraction. Figure 11 shows the differences arising then from the various odors, as revealed by the ensuing statistical difference maps. Clear positive and negative regions of differential activation arise among the regions elicited by the different odors, at 60 ppm odor concentrations. Given the relatively small number of samples included in these second-level analyses, these results were also evaluated using SnPM (https://github.com/SnPM-toolbox), a non-parametric statistical framework. The outcome of these second-level SnPM analyses are presented in the Supplementary Information, Figure S3, which shows similar difference maps as Fig. 11 except at the *p* < 0.01 limit, where the appetitive-neutral differences are less certain.

## Discussion and conclusions

This study explored the potential that spatiotemporal encoding single-shot modalities applied at ultrahigh fields, can open to capture the activity of the OB under different olfactory stimulations. The signal and activation maps obtained at 15.2 T were unambiguous; despite relying on similar anesthesia protocols and despite SPEN’s generally longer echo times, they appeared significantly stronger than recent 7 T results where functional activation reached ≤ 1%^28^. We ascribe this largely to the performance of these measurements at ultrahigh fields, and to the ca. quadratic increase in the BOLD signal activation that—even when targeting small capillaries as in highly refocused experiment—is expected to arise with field strength^40–43^. These results are thus consistent with activations observed at 17.2 T by alternative, multi-shot ultrahigh field MRI modalities^20^. Working at ultrahigh fields, however, highlights EPI’s limitations to deal with field inhomogeneities, particularly when targeting regions like the OB sited close to air interfaces. GE and SE EPI images can still be obtained from this region, but artifacts and distortions would make it difficult to pinpoint the regions being activated in such small organ. SPEN MRI incorporating various levels of T2* refocusing alleviates this restriction, providing sufficient anatomical resolution in the fMRI data to highlight the discriminated response against appetitive/neutral/aversive stimuli in the central glomeruli and in the external plexiform regions—with the latter showing a stronger response for aversive/predator odors and the former for appetitive ones (Figs. 10, 11). The activation patterns then elicited are consistent with 11.7 T results of a blood volume-weighted MRI study^44^, in which similar odor-specific spatial activation patterns were reported; such previous studies, however, relied on the injection of super-paramagnetic contrast agent for delivering their information based on FLASH MRI, rather than intrinsic activation. The good ensuing agreement highlights the complementarity between these two functional approaches. Ongoing work focused on improving the functional response described in this study is in progress, in the expectation that improved paradigms will enable us to explore subtler stimuli. Of particular interest would be assaying whether the effects of pheromones can be discerned by fMRI on naïve rodents, as well as exploring fMRI on mice possessing dysfunctional olfactory systems^45^. Additional experiments extending the influence of the assayed paradigm further upstream in the brain, also need to be developed for providing a more comprehensive picture of olfaction. The relatively slow nature of the fMRI SPEN approaches here used, resulting from their use of non-selective (chirped) inversion pulses, might be ill-suited for such extended volume coverage; these cases might benefit from a hybrid approach where SPEN is utilized to target the more challenging reasons, while slice-selective EPI targets other, more amenable brain regions. Still, while progress remains to be made, the methodology demonstrated in this study holds promise for future olfactory fMRI studies in the OB of mammals (including humans), as well as in other brain regions suffering at high fields from large susceptibility artifacts.

### Supplementary Information

Supplementary Information.

## Data Availability

The raw data supporting the findings of this study as well as the processed images resulting from it, are available from the corresponding author upon request.

## Code availability

The codes used to process the data and analyze the images of this study, as well as the MRI pulse sequences that were used, are available from the corresponding author upon request.
