# Author Correction: Functional MRI of murine olfactory bulbs at 15.2 T reveals characteristic activation patterns when stimulated by different odors

**DOI:** 10.1038/s41598-023-42524-0

**Published:** 2023-09-20

**Authors:** Odélia Chitrit, Qingjia Bao, Aoling Cai, Silvia Gabriela Chuartzman, Noga Zilkha, Rafi Haddad, Tali Kimchi, Lucio Frydman

**Affiliations:** 1 Department of Chemical and Biological Physics, Weizmann Institute of Science, Rehovot, Israel; 2 Department of Brain Sciences, Weizmann Institute of Science, Rehovot, Israel; 3 Innovation Academy for Precision Measurement Science and Technology, Chinese Academy of Sciences, Wuhan, China; 4 The Gonda Multidisciplinary Brain Research Center, Bar-Ilan University, Ramat‑Gan, Israel

Correction to: *Scientific Reports* 10.1038/s41598-023-39650-0, published on 16 August 2023

In the original version of this Article, Tali Kimchi was omitted as a corresponding author. Correspondence and requests for materials should also be addressed to tali.kimchi@weizmann.ac.il

Also, The Funding section in the original version of this Article was omitted. The Funding section now reads:

“Support from the Minerva Foundation (Germany), the Israel Science Foundation (grants 3594/21, 2141/21 and 1874/22) and the Perlman Family Foundation, are gratefully acknowledged. OC is the recipient from an Israel Ministry of Absorption fellowship. LF holds the Bertha and Isadore Gudelsky Professorial Chair and Heads the Clore Institute for High-Field Magnetic Resonance Imaging and Spectroscopy (Weizmann Institute) whose support is also acknowledged”

The original Article has been corrected.

