# Supplementary material for: Functional MRI of murine olfactory bulbs at 15.2T reveals characteristic activation patterns when stimulated by different odors
Source: Sci Rep. 2023 Aug 16;13:13343. doi: 10.1038/s41598-023-39650-0 (PMC10432392; doi:10.1038/s41598-023-39650-0)
Supplement: Supplementary file 1 — Supplementary Information. [file 41598_2023_39650_MOESM1_ESM.pdf]

Supplementary information for

Functional MRI of murine olfactory bulbs at 15.2T reveals characteristic activation patters when stimulated by different odors

Odélia Chitrit[1], Qingjia Bao[1,3], Aoling Cai[3], Silvia Gabriela Chuartzman[2], Noga Zilkha[2], Rafi Haddad[4], Tali Kimchi[2] and Lucio Frydman[1,*]

Departments of [1]Chemical and Biological Physics and [2]Brain Sciences, Weizmann Institute of Science, Rehovot, Israel. [3]Innovation Academy for Precision Measurement Science and Technology, Chinese Academy of Sciences, Wuhan, China. [4]The Gonda Multidisciplinary Brain Research Center, Bar-Ilan University, Ramat-Gan, Israel

Supplementary Table S1: Main acquisition parameters of the fMRI experiments

Parameter	2D SPEN	2D SE EPI
Repetition time (ms)	1000	1000
Echo Time (ms)	26.03	26.78
Overall acquisition time	23 min	23 min
Interleaved segments	1	1
Slice thickness (mm)	0.8	0.8
Field of View (mm)	10.4x7.5	11x10
Matrix size	96x70	96x96
Nominal resolution (µm)	108x107	115x104
Readout bandwidth (kHz)	450	400
2nd dimension (Phase, SPEN bandwidths – kHz)	13.5	3.6
SNR of OB in image	30 ± 10	25 ± 10
SPEN's pulse time-bandwidth product	100	-
SPEN's chirp duration (ms)	7.4	-
SPEN's PR T2 delay (ms)*	4	-

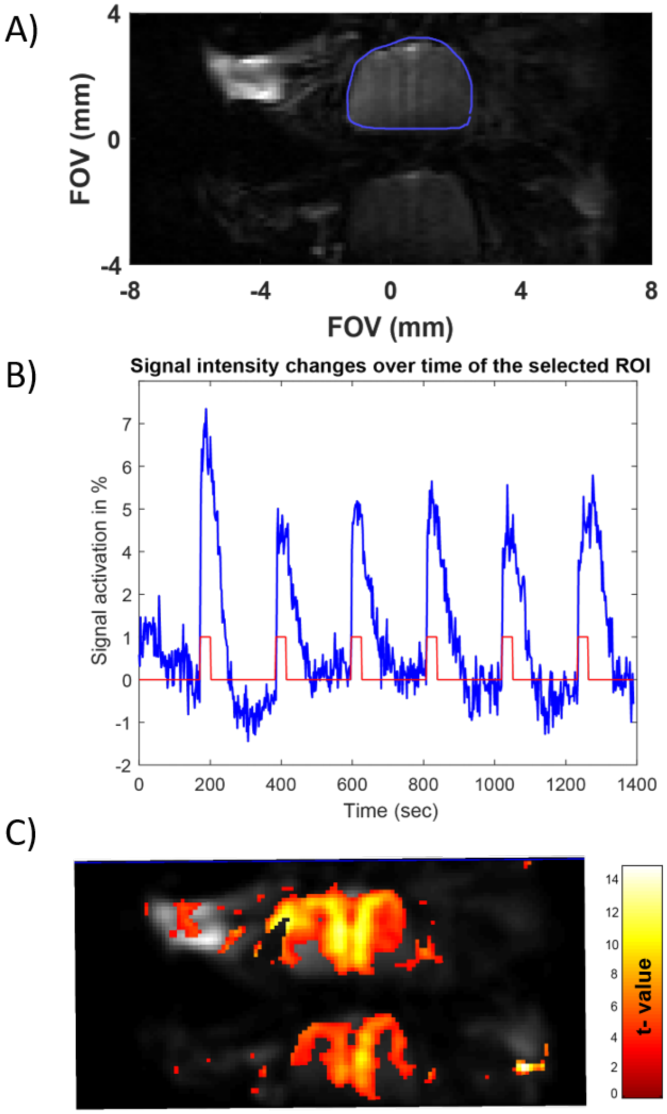

Supplementary Figure S1: fMRI experiment collected with SE-EPI. A) Outcome of the SE-EPI acquisition. B) Signal activation arising from the circled region of interest in A), and its paradigm (180sec air / 30sec odor)x6. C) SPM activation map for p<0.001.

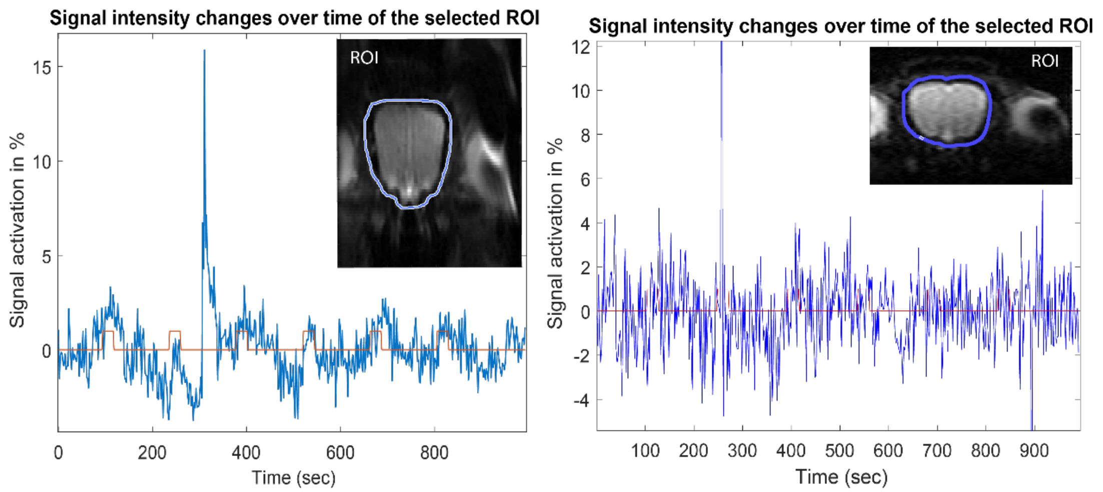

Supplementary Figure S2: fMRI control experiments carried out without any odor: (Left) SE-EPI acquisition; (Right) Fully-refocused SPEN acquisition. The paradigm used was as in the actual olfaction experiments, switching between an air line and an odor line carrying solely the carrier gas as indicated by the red traces. Notice the pseudo-activation yielded in the SE-EPI case, presumably due to this sequence's higher sensitivity to small motions. Large spikes correspond to large animal motions.

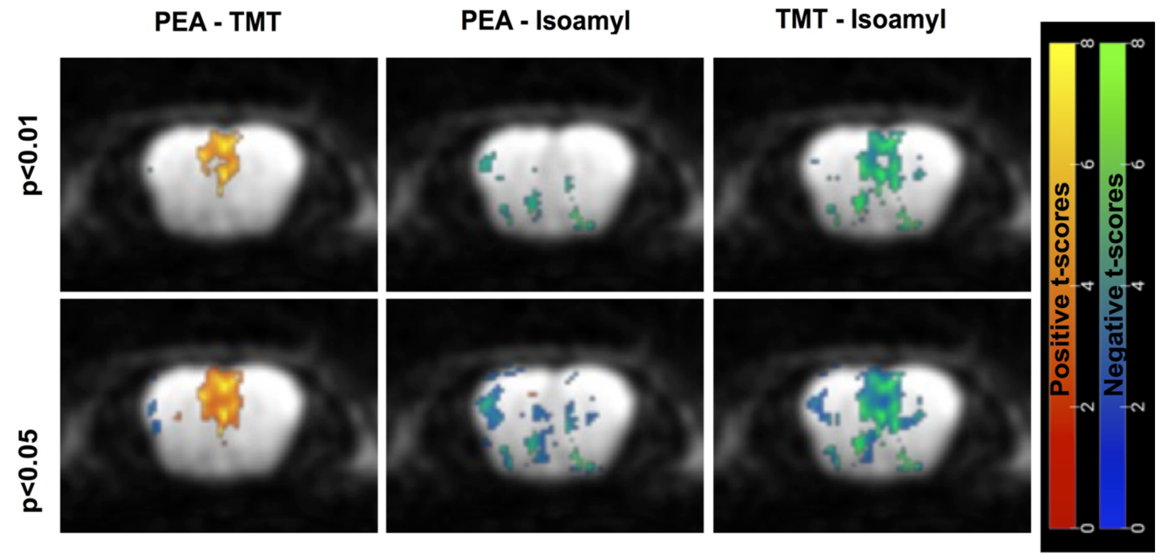

Supplementary Figure S3: Idem as Figure 11 in the main text, except that upon processing the data with a non-parametric (rather than parametric) statistical framework. Other than for the center panel images, there is a close correlation between the information arising from the two sets.
